# Rational use of medicines: Achievements and challenges

**DOI:** 10.4103/0253-7613.64486

**Published:** 2010-04

**Authors:** Vijay Thawani

**Affiliations:** Vijay Thawani, Nagpur, India E-mail: vijaythawani@rediffmail.com

The issue of rational use of medicines (RUM) has been throbbing since decades and the essential medicine (EM) concept has been pulsating for almost a quarter century. The essential medicine list (EML), accessibility, affordability and availability of EM have been some of the important issues in RUM. The critics broadcast that these issues are favorite past time of the policy makers and only serve the agenda of their conclaves. Time, that we analyse their impact on the medicine scenario and various stakeholders.

The RUM in any country is influenced by its national medicine policies. It is unfortunate that the need for RUM is greatest in poorer economies where financial resources are scarce and pressing needs multiple. This sacrifices the attention and resource allocation for RUM at the altar of policy makers resulting in continual suffering of their populations because of lopsided preferences. In the countries, which have been convinced about the need of RUM, effective steps have been taken for its promotion. Most of the economically developed and many developing nations have a medicine policy and their EML is regularly updated. Many countries have adopted the policies of generic use, teaching and training about EM concept at undergraduate (UG) level, pharmacovigilance programs and prescription audits, all contributing to the greater goal attainment of RUM.

Having got the opportunity to attend WHO-sponsored training in an international RUM course, we were sensitized to study and promote RUM. That was a great push, which allowed us to peep at the wider problem of use, misuse and irrational use of medicines. With strong conviction need for such training to others, some of us became facilitators for RUM and have been training for the last many years. The involvement of international participants and their inclination to stay connected on a platform where RUM-related issues can be freely discussed without censorship, led to the constitution of a net group of motivated and concerned – NetRUM, which has been growing ever since and has discussed more than 150 hot topics related to RUM.

The accessibility, availability and affordability across several countries have been researched by WHO and Health Action International (HAI). In their surveys, interesting facts came up from developing countries including India, where it was found that though the medicine prices were lower as compared to the international prices, the affordability was poor. In most developing countries, the patients pay out of pocket for their medicines, unlike in developed economies where medical insurance or co-pay systems pay for the health costs. That in our part of the world, the lowest priced generics are as expensive as or more expensive than the originator brand has been disturbing. The high mark-ups on medicines have to be seen to be believed in India. Charging exorbitant profits is called profiteering, which is detrimental for RUM. This continues to flourish and the citizenry has no effective pressure groups of activists who can fight for the common cause of RUM.

The RUM also includes correct prescribing, dispensing and adherence. The establishment of Drugs and Therapeutic Committees (DTCs) in hospitals is a step forward in the direction of correct prescribing. Stray reports of educational, managerial and regulatory strategies being adopted offer a welcome ray of hope. Apart from improving medicine availability, financial management, monitoring promotion, prescribing behavior and a sound medicine policy, a well-evidenced compelling need is felt for public education in RUM. The public education strategies provide momentum in raising awareness, creating knowledge, empowering community, change attitude and practices for RUM. In a global survey of the different projects going on to create a database and to identify the strengths and weaknesses of the ongoing activities, it was found that most interventional studies lacked in proper monitoring and evaluation of their projects. The sustained effect of such interventions was not clear and replicability in other situations/countries was a matter to be undertaken with caution.

Orientation of pharmacy students towards RUM monitoring and evaluation and good pharmacy practice supplements RUM. Medicine promotion directly to the consumers and to the prescribers is another area where planning of interventional research gives fruitful results. The current educational initiatives for pharmacy and medical students about medicine promotion are abysmal. Pharmacovigilance is still in nascent stage and yet to be taken seriously in developing economies. Voluntary reporting of ADRs by doctors, pharmacists and patients should be encouraged. Need is also there for the interventional studies in medicine use in health and dispensing facilities.

In post-Independence era, the quality assurance and safety of medicines have shown improvement in India but much remains to be done in quality generic production. The EML for pediatric medicines has recently initiated in India with patronage of WHO. Generic prescribing needs to be enforced with great vigor. The regulations need to be amended so that a qualified pharmacist can substitute the branded prescription with a generic one. More Janaushadhi generic pharmacies need to be opened. The access to EM in SEARO countries is still low (8.7–15%). Measuring access to EM is a good indicator for RUM in the country. Since the public health services are with State Governments, the successful example of Tamil Nadu and Delhi should be emulated in medicine procurement.

Several strategies have been suggested in WHO policy perspective for RUM as given below:

1. Evidence-based standard treatment guidelines (STG);
2. EML based on treatments of choice;
3. DTC in hospitals;
4. Problem-based training in pharmacotherapy in UG teaching;
5. CME as a licensure requirement;
6. Independent medicine information;
7. Supervision, audit and feedback;
8. Public education;
9. Avoidance of perverse financial incentives and
10. Appropriate and enforced drug regulation.

While some of the above may have received attention, others remain to be attended to. Two International Conferences on Improving Use of Medicines (ICIUM) have so far been held with WHO support and the third is planned in 2011 in Egypt. The WHO makes efforts to ensure that global experiences are widely shared and discussed during these meets.

However, it is disturbing to note that the WHO and World Trade Organization (WTO), both UN organizations, are at loggerheads. While one aims for 'Health for all', the other has been instrumental in compelling adoption of the policies of 'Wealth for few' in intellectual property rights (IPR). It remains to be seen how, in future, the pragmatic world order will change this in favor of the marginalized.

Monitoring and using the collected information to develop, implement and evaluate strategies to change the behavior of inappropriate medicine use are fundamental to the success of RUM. Change is possible, resistance can be overcome, but 'will' to change is required. Let us just do it!

